# Breaking the cancer code: a novel DNA minicircle to disable STAT3 in ovarian cancer cells SKOV3

**DOI:** 10.3389/fphar.2025.1673427

**Published:** 2025-09-15

**Authors:** Adina-Gabriela Vasilescu, Andrei-Mihai Vasilescu, Livia Elena Sima, Natalia Baran, Ștefan-Eugen Szedlacsek

**Affiliations:** ^1^ Department of Enzymology, Institute of Biochemistry of the Romanian Academy, Bucharest, Romania; ^2^ Department of Molecular Biology of the Cell, Institute of Biochemistry of the Romanian Academy, Bucharest, Romania; ^3^ Department of Hematology and Central Hematology Laboratory, Inselspital, Bern University Hospital, University of Bern, Bern, Switzerland; ^4^ Department of Internal Medicine, University of South Dakota, Sioux Falls, SD, United States

**Keywords:** STAT3, DNA minicircle, ovarian cancer, decoy inhibitor, apoptosis

## Abstract

**Introduction:**

Ovarian Cancer remains a significant global health concern, with high mortality rates, largely due to late-stage diagnosis and limited treatment options. These extrinsic factors are driven or exacerbated by intrinsic mechanisms such as persistent activation or upregulation of Signal Transducer and Activator of Transcription 3 (STAT3). STAT3 promotes tumor growth, inhibits apoptosis, accelerates angiogenesis and metastasis, facilitates immune evasion, and contributes to chemoresistance. Consequently, STAT3 activation fosters an aggressive ovarian cancer phenotype, contributing to treatment failure, poor prognosis and low survival rates, highlighting the urgent need for novel, safe, effective and affordable STAT3-targeted therapeutic strategies. In this study, we developed a novel double-stranded DNA minicircle (mcDNA) inhibitor, designed to act as a decoy for STAT3, preventing its binding to target gene promoters.

**Methods:**

Utilizing the SKOV3 ovarian cancer cell line, we evaluated the effects of our inhibitor *in vitro* on cell viability through MTS assay, its apoptotic and necrotic effects using flow cytometry and the expression modulation of downstream STAT3-regulated genes, assayed through RT-qPCR and Western blot analysis.

**Results:**

We demonstrate that anti-STAT3 mcDNA significantly reduces the viability of SKOV3 cells at low nanomolar concentrations, while sparing the control group. The effect observed was dose-dependent. Mechanistically, anti-STAT3 mcDNA induces apoptosis and necrosis in treated cells, also revealing a certain dose-dependency, while also decreasing cell proliferation. Finally, our inhibitor significantly downregulates STAT3-dependent anti-apoptotic genes *MCL1* and *PIM1*.

**Conclusion:**

These findings suggest that anti-STAT3 mcDNA is a promising, effective and specific candidate for targeted STAT3 inhibition in SKOV3 ovarian cancer cells, warranting further validation in ovarian cancer, *in vivo* exploration and potential application in other types of malignancies, where STAT3 acts as an oncogenic factor.

## 1 Introduction

Ovarian cancer (OC) is one of the most aggressive gynecological malignancies worldwide, primarily due to its silent growth and rapid progression (National Cancer Institute, 2025). Annually, there are over 200,000–300,000 newly diagnosed cases globally (World Cancer Research Fund, 2025). Despite this high incidence, the prevalence appears low, mainly due to its high mortality rate, late-stage diagnosis, and limited therapeutic options for disseminated disease, which lead to short survival durations. A key molecular driver underlying these clinical features is the persistent activation and upregulation of STAT3 signaling. Aberrant STAT3 activity has been associated with OC progression, metastasis, chemoresistance, metabolic reprogramming, angiogenesis, immune evasion and poor prognosis in OC patients (Silver et al., 2004; Huang et al., 2022; Gao et al., 2021).

The JAK/STAT3 pathway is a fundamental signaling cascade activated by cytokines and growth factors that coordinates major cellular processes (Kiu and Nicholson, 2012). Upon ligand binding, such as Interleukin 6 (IL-6) or Epidermal Growth Factor (EGF), to their respective receptors, Janus kinases (JAKs) become fully activated and further phosphorylate the cytoplasmic domain of the receptor. This phosphorylation creates docking sites for the Signal Transducer and Activator of Transcription 3 (STAT3) (Jatiani et al., 2010). STAT3, once recruited to these sites, is directly phosphorylated by JAKs at Tyr705, forms dimers via its Src Homology 2 (SH2) domain, and is translocated to the nucleus, where it binds to downstream genes (Schindler et al., 2007). The described binding of STAT3 to target genes takes place through recognition of specific elements in their promoter regions, known as Interferon-gamma activation sites (GAS), via the DNA-binding domain (DBD) of STAT3 (Martincuks et al., 2016). In physiological conditions, STAT3 is considered essential both in early development (Azami et al., 2025) and adult differentiated tissues, where it regulates cell growth, survival, proliferation, immune response and stemness (Levy and Lee, 2002).

Persistent STAT3 hyperactivation may result from excessive cytokine or growth factor signaling, increased JAK kinase activity, or the loss of STAT3 negative regulatory mechanisms (Tolomeo and Cascio, 2021). Aberrant constant activation of STAT3 has been observed in several human cancers, including ovarian, head and neck, breast, or lung cancers, driving multiple oncogenic pathways (Sonnenblick et al., 2012; Harada et al., 2014; Geiger et al., 2016; Saini et al., 2017). It has been shown to upregulate cyclin D1 and c-Myc, promoting cell-cycle progression and proliferation, respectively (Leslie et al., 2006; Shirogane et al., 1999). Notably, STAT3 induces the expression of anti-apoptotic and pro-survival genes such as *Bcl-2, Bcl-xL, survivin, PIM1, or MCL1*, as well as extracellular matrix modulators like matrix metalloproteinases (MMPs), thereby supporting tumor growth, metastasis and chemoresistance (Shirogane et al., 1999; Kanda et al., 2004; Yu and Jove, 2004; Tsareva et al., 2007).

Furthermore, STAT3 suppresses death receptor signaling pathways, such as Fas, allowing cells to evade apoptosis (Barré et al., 2007). Moreover, it promotes angiogenesis by stimulating the expression of vascular endothelial growth factor (VEGF) and enhancing endothelial tube formation, contributing to the prominent vascularity within tumors (Pepper et al., 1992; Niu et al., 2002).

Given these observations, there is a pressing clinical and scientific need to develop effective STAT3 inhibitors to establish foundational therapeutic strategies. Various molecular approaches have been developed to inhibit STAT3 signaling in cancer. Notably, several studies have focused on designing and synthesizing peptides (such as PY*LKTK) or small molecules (including STATTIC, OPB-31121, C188, or Napabucasin) that target the SH2 or DNA-binding (DBD) domains of STAT3, effectively abrogating its signaling. Yet, these molecules generally exhibit rather limited success in clinical use due to toxic side effects, unsatisfactory pharmacokinetic properties, high levels of nonspecific activity, or limited therapeutic efficacy (Turkson et al., 2001; Schust et al., 2006; Brambilla et al., 2015; Lewis et al., 2015; Shitara et al., 2019). Interestingly, curcumin, a natural compound, has been reported to inhibit STAT3 signaling by downregulating the expression of target genes, such as *BCL-2* and *survivin*, in pancreatic cancer (Glienke et al., 2010). However, the lack of a comprehensive understanding of curcumin’s mechanism of action and limitations for rational drug design hinder its development as a therapeutic agent.

Nucleic acid-based inhibitors targeting STAT3 expression represent another class of therapeutic agents that have been explored. Antisense oligonucleotides, which are short 12–25 nucleotides-long DNA molecules, were designed complementary to STAT3 mRNA in order to disrupt the translation of the STAT3 protein (Hong et al., 2015). However, due to their single-stranded DNA structure, they need extensive chemical modifications to protect against nuclease degradation. siRNA molecules targeting STAT3 have demonstrated promising effects, such as reduced expression levels of STAT3-regulated proteins, reduced proliferation, and increased apoptosis in various types of cancer, including ovarian carcinoma (Sun et al., 2015; Han et al., 2013). Nevertheless, stability issues and off-target effects remain the greatest challenge for these approaches. Foundational work has been done with inhibitors named Oligodeoxynucleotide Decoys (ODN-decoys) (Crinelli et al., 2002), which have been extensively studied in different cancer models. Of note, promising results were yielded in OC by the STAT3 ODN-decoy (Liu et al., 2014). The latter are double-stranded molecules of 15 base pairs that mimic, through their sequence, the specific binding site within the promoter region of STAT3-regulated genes. This design allows them to act as competitors for the DBD of STAT3.

By diverting STAT3 binding, these decoys inhibit the expression of downstream anti-apoptotic and pro-survival genes (Johari et al., 2020). This STAT3 ODN-decoy has been tested in various cancer cell lines, where it displayed promising effects, such as apoptosis induction, diminution of cancer cell proliferation, and reduction in *Bcl-xL* and *cyclin D1* gene expression (Zhang et al., 2007). However, when further tested in a phase 0 clinical trial (Sen et al., 2012), it failed to achieve the same inhibitory effect on cancer growth, most likely due to fast degradation by nucleases present in the serum. To improve stability, cyclic molecules were developed using hexaethylene glycol linkers to seal the ends previously exposed to nucleases. This modified decoy appeared more stable and capable of reducing tumor growth in xenograft models of non-small cell lung cancer and head and neck cancer (Njatcha et al., 2018; Sen et al., 2014).

This work demonstrates, for the first time, the generation and application of double-stranded DNA minicircles bearing three GAS-like motifs as inhibitors of STAT3 *in vitro*, using the highly chemoresistant SKOV3 cell line model for ovarian cancer (Ishiguro et al., 2016). To this end, we synthesized an anti-STAT3 minicircle DNA (anti-STAT3 mcDNA) and a negative control minicircle, which is similar in structure, but contains mutated GAS-like sequences (mock mcDNA).

We believe that the advantages of our minicircles include: (i) the presence of 3 GAS-like motifs on the same molecule maximizing the chance of DBD-STAT3 binding; (ii) a closed circular structure, which confers increased stability and resistance to nuclease degradation; (iii) a potentially long half-life when administered *in vivo*; and (iv) ease and cost-effectiveness of production, as they are simple DNA molecules with no chemical modifications other than 5′-phosphorylation of the precursor oligonucleotides. We anticipate that these features will translate into a highly efficient inhibitory strategy against STAT3 in OC, warranting further studies in STAT3-dependent cancers.

## 2 Materials and methods

### 2.1 Cell culture

The human ovarian carcinoma cell line SKOV3 (stored in our laboratory; American Type Culture Collection–ATCC, ref. no. HTB-77) was used for all cell-based experiments. The cells were confirmed to be mycoplasma-free and grown in RPMI 1640 medium (PAN Biotech, with L-Glutamine, Cat no. P04-16500) supplemented with 10% heat-inactivated fetal bovine serum (FBS), MEM Non-Essential Amino-Acids (Gibco, Cat no. 11140–050) and antibiotics (50 IU/mL penicillin and 50 μg/mL streptomycin). Cultures were maintained at 37 °C in a humidified atmosphere with 5% CO_2_. Cells were used between passages 3 and 9. For experiments involving DNA minicircles treatment, cells were resuspended and seeded in the same medium without antibiotics.

### 2.2 Anti-STAT3 and mock oligonucleotides

The 5′ phosphorylated sense and antisense strands of 95 nucleotides, required to produce the anti-STAT3 and mock minicircles, were ordered from GenScript Biotech (Netherlands). The sequences of the anti-STAT3 strands were designed to contain three equally spaced GAS-like motifs (in bold): 5′ GGG​CGC​ATA​TTC​GGA​CGG​A**TT​CCC​GTA​A**TG​CCT​TAT​ACT​GCG​ACG​ATA​TCA​**TTC​CCG​TAA**​TAG​AGT​TCG​CTA​CTA​GCT​GTC​GA**T​TCC​CGT​AA**C​CC 3′ and 5′ GGG​**TTA​CGG​GAA**​TCG​ACA​GCT​AGT​AGC​GAA​CTC​TA**T​TAC​GGG​AA**T​GAT​ATC​GTC​GCA​GTA​TAA​GGC​A**TT​ACG​GGA​A**TC​CGT​CCG​AAT​ATG​CGC​CC 3’. The mock sequences were designed to bear three equally distant scrambled GAS-like motifs (in bold): 5′ GGG​CGC​ATA​TTC​GGA​CGG​A**TA​GCC​TTA​A**TG​CCT​TAT​ACT​GCG​ACG​ATA​TCA​TAG​CCT​TAA​TAG​AGT​TCG​CTA​CTA​GCT​GTC​GA**T​AGC​CTT​AA**C​CC 3′ and 5′ GGG**​TTA​AGG​CTA**​TCG​ACA​GCT​AGT​AGC​GAA​CTC​TA**T​TAA​GGC​TA**T​GAT​ATC​GTC​GCA​GTA​TAA​GGC​A**TT​AAG​GCT​A**TC​CGT​CCG​AAT​ATG​CGC​CC 3’. For the circularization of these strands, several short DNA oligos (14–21 nucleotides), called splints, were also ordered from GenScript Biotech. Each splint was designed to hybridize with one of the above-mentioned oligonucleotides to facilitate ligation. The sequences for the anti-STAT3 splints were: 5′ GCGCCCGGGTTACG 3′ and 5′ CCG​TAA​CCC​GGG​CGC​ATA​TTC 3’. For the mock splints, the sequences were: 5′ GCGCCCGGGTTAAG 3′ and 5′ CCT​TAA​CCC​GGG​CGC​ATA​TTC 3’.

### 2.3 Double-helical complex splint-assisted enzymatic cyclization of oligonucleotides

Each oligo and its corresponding splint (1 µM oligo:3 µM splint) were combined in nuclease-free water to a final solution volume of 1 mL and T4 DNA Ligase Reaction Buffer (NEB, final concentrations 50 mM Tris-HCl pH 7.5, 10 mM MgCl2, 1 mM ATP, 10 mM Dithiothreitol–DTT). To ensure the efficiency of the reaction and to avoid linear ligation, which results in unwanted multimer formation, the oligonucleotides utilized were very diluted in the ligation reaction. The mixture was incubated in a thermoblock at 90 °C for 10 min with inversion every 2 min, then allowed to slowly cool down to room temperature (RT, 25 °C) to facilitate annealing. Subsequently, DTT (Promega; Cat no V3155) was added to a final concentration of 10 mM, ATP (NEB; Cat no P0756L) to a final concentration of 100 μM and 3 U/µL of Hi-T4™ DNA Ligase (NEB, Cat no M2622S) to a final volume of 1 mL. The ligation reaction was incubated at RT for 16 h to achieve circularization.

### 2.4 Isolation of circular single-stranded molecules, annealing and enzymatic verification

Following the ligation incubation, T4 DNA Ligase was inactivated at 65 °C for 10 min, then the samples were immediately placed on ice. The reaction volume was reduced using a speed vacuum concentrator, and the DNA was mixed with 6X Loading Dye Purple (NEB; Cat no B7024S). The samples were subjected to electrophoresis and run on a 3.5% agarose gel at 55V for 1 h and 30 min. DNA bands corresponding to the single-stranded minicircles were excised and purified using Zymoclean Gel DNA Recovery Kit (Zymo Research, Cat no D4001T).

The concentration and the purity of the recovered DNA were determined with a NanoDrop™ 2000 (Thermo Scientific). The purified single-stranded circles, the precursors to the anti-STAT3 mcDNA, were annealed to form double-stranded minicircles. Annealing was performed in buffer containing 10 mM Tris-HCl (pH 8.0), 50 mM NaCl, and 1 mM MgCl_2_. The mixture was incubated at 95 °C for 5 min, then slowly cooled down to RT. The same procedure was applied to the mock minicircle single strands. The circularity of the double-stranded anti-STAT3 minicircle and mock minicircle DNA was verified by enzymatic digestion at 37 °C for 1 h using SmaI alone, EcoRV alone, or both enzymes simultaneously. Following digestion, samples were immediately placed on ice, mixed with Loading Dye Purple, and analyzed on a 3.5% agarose gel, at 55V for 1 h and 30 min. These steps were repeated multiple times to obtain sufficient quantities of minicircles for subsequent cell biology experiments.

### 2.5 Determination of anti-STAT3 mcDNA interaction with STAT3 protein through electrophoretic mobility shift assay (EMSA)

Professor Eyal Arbely from the Department of Chemistry, Ben-Gurion University of the Negev, Beer-Sheva 8410501, Israel, kindly provided a plasmid encoding STAT3, which was used in our lab to obtain the STAT3 protein, as previously described (Belo et al., 2019). Anti-STAT3 mcDNA, or mock mcDNA were incubated with STAT3 protein for 1 h, at 25 °C, in an interaction buffer consisting of 10 mM Tris (pH 7.8), 100 mM NaCl, 0.1 mM DTT and 1 mM EDTA, in varying DNA:protein molar ratios. For anti-STAT3 mcDNA, the tested molar ratios were 1:0, 1:0.75, 1:1.5, 1:2, 1:3 and 1:4. For mock mcDNA, the ratios were 1:0, 1:3 and 1:4. After the incubation, the samples were mixed with Loading Dye Purple and run on a 2.5% agarose gel, at 80V for 40 min.

### 2.6 Transfection of anti-STAT3 and mock mcDNA molecules

Transfection was performed using Lipofectamine™ 3000 Transfection Reagent (Invitrogen, Cat no L3000001). One day prior to transfection, SKOV3 cells were seeded to achieve 70%–90% confluence at the time of transfection. Cells were transfected with either anti-STAT3 mcDNA or mock mcDNA using a DNA (µg) to Lipofectamine 3000 (µL) ratio of 1:1. Six hours post-transfection, the culture medium was replaced with fresh complete RPMI medium without antibiotics. The cells were incubated for 48 h post-transfection. The concentrations of anti-STAT3 mcDNA and mock mcDNA used for the MTS assay were 0 nM, 1.56 nM, 3.12 nM, 6.25 nM, 12.5 nM, 25 nM, 50 nM, and 100 nM. For flow cytometry analysis, transfection concentrations of 5 nM, 10 nM, and 20 nM were employed. For RT-qPCR and Western blot experiments, a concentration of 10 nM was used.

### 2.7 MTS assay

Cell viability was assessed using the CellTiter 96^®^ AQueous One Solution Cell Proliferation Assay (Promega; Cat no G3580). Following transfection with anti-STAT3 mcDNA or mock mcDNA, the culture medium was replaced with complete medium (without antibiotics) mixed with the MTS reagent at a proportion recommended by the manufacturer. Cells were incubated at 37 °C, and absorbance was measured at 490 nm every 15 min using the FLUOstar Omega 96-well plate reader (BMG Labtech). The medium-MTS mixture without cells was used as blank. Data were expressed as the absorbance values over concentration to assess cell viability.

### 2.8 Flow cytometry analysis for transfection efficiency

We performed transient transfection using either Polyethylenimine (PEI; Merck, Cat no 408727) or Lipofectamine™ 3000 Transfection Reagent. One day prior to transfection, SKOV3 cells were seeded 1 × 10^5^ cells *per* well in a 12-well plate. Transfections were carried out with different DNA:transfection reagent ratios. For Lipofectamine 3000, the ratios were 1:1 and 1:2 (µg DNA: µl reagent); while for PEI, the ratios were 1:3 and 1:5 (µg DNA: µl reagent).

To determine transfection efficiency, cells were washed with PBS, trypsinized, and collected by centrifugation at 1500 rpm for 5 min at 4 °C. Pelleted cells were resuspended in FACS buffer (2% FBS in PBS) and counted, then adjusted to a final density of 1 × 10^6^ cells/mL. GFP expression in transfected SKOV3 cells was detected via flow cytometry using the 488 nm laser. Untreated, unstained cells and cells treated with Lipofectamine-only or PEI-only served as negative controls.

### 2.9 Apoptosis evaluation through flow cytometry

At 48 h post-transfection, cells were washed with cold PBS, collected into FACS tubes, and centrifuged at 1500 rpm for 5 min at 4 °C. Cell pellets were resuspended in Annexin buffer (10 mM HEPES pH 7.4, 2.5 mM CaCl_2_, 140 mM NaCl) and counted with a hemocytometer. The cell suspension was diluted in the same buffer to a final density of 1 × 10^6^ cells/mL. Next, 100 µL aliquots were incubated with 5 µL of Annexin V-FITC (BioLegend, Cat no 640905) and 1 µL (final concentration 5 μg/mL) of Propidium Iodide (PI, BioLegend, Cat no 421301) for 15 min in the dark, at RT. Untreated, unstained cells served as negative controls, while cells treated with Cisplatin (20 μM, for 24 h), stained with Annexin V-FITC, and cells treated with Triton X-100 (0.02%, for 15 min), stained with PI, served as the single-stain positive controls for compensation purposes. After staining, cells were diluted with 400 µL Annexin buffer, and 10,000 events *per* condition were acquired on a BD FACSVerse™ cytometer (BD Biosciences). Excitation was performed with a 488 nm laser, detecting FITC and PI fluorescence. Autofluorescence was determined using unstained controls. Data analysis was conducted using the Cytobank platform (Kotecha et al., 2010). The flow cytometry dot plots and histograms were gated to identify singlets (FSC-A vs. FSC-H) and to differentiate viable, apoptotic, and necrotic populations based on Annexin V and PI fluorescence.

### 2.10 RT-qPCR assay

Total RNA was extracted using the RNeasy kit (QIAGEN, Cat no 74104). RNA concentration and quality were determined with a NanoDrop™ 2000 spectrophotometer. Reverse transcription was performed with 1 µg of RNA using the Maxima First Strand cDNA Synthesis Kit for RT-qPCR with dsDNase (Thermo Scientific; Cat no K1641), following the manufacturer’s instructions for incubation times and temperatures. Quantitative PCR (qPCR) was carried out using 1 µL of cDNA with gene-specific primers and the SensiFAST™ SYBR^®^ No-ROX Kit (Bioline, Cat no BIO-98005). The target genes were *MCL1* and *PIM1*, and normalization was performed using *GAPDH* as the housekeeping gene (Supplementary Table S1). Negative controls lacking reverse transcriptase were included for all three genes. Reactions were performed on a Rotor-Gene 6000 (QIAGEN). All primers were synthesized by Eurofins Genomics (Germany). The qPCR protocol consisted of an initial enzyme activation and denaturation at 95 °C for 2 min, followed by 40 cycles of: 95 °C for 5 s (denaturation), 63 °C for 10 s (annealing), and 72 °C for 20 s (elongation). A final extension step at 72 °C for 2 min was performed. Melting curve analysis was conducted from 72 °C to 95 °C, increasing by 1 °C per second. Fluorescence signals were acquired during the elongation step in the green channel. Data analysis was performed using the Rotor-Gene Q Series Software v2.3.5 (QIAGEN) for comparative quantitation analysis (Warton et al., 2004).

### 2.11 Western blot analysis

SKOV3 cells were lysed directly in RIPA buffer supplemented with protease and phosphatase inhibitors (cOmplete™ EDTA-free Protease Inhibitor Cocktail, Roche, Cat no 4693132001), 1 mM sodium orthovanadate, 1 mM Phenylmethylsulfonyl fluoride (PMSF), and 5 mM iodoacetic acid. Lysates were incubated at 4 °C with gentle shaking for 40 min, then centrifuged at 20,000 x g for 40 min at 4 °C. The supernatant was collected, and protein concentrations were determined using the Pierce™ BCA Protein Assay Kit (Thermo Fisher; Cat no 23225). Absorbance was measured at 562 nm after a 30-min of incubation at 37 °C using a FLUOstar Omega plate reader (BMG Labtech), with lysis buffer as the blank. Equal amounts of protein were mixed with Laemmli sample buffer, boiled for 5 min at 95 °C, and separated by SDS-PAGE on either in-house prepared Tris-glycine or precast gels (Bio-Rad). Proteins were transferred in a wet system to PVDF membranes for 1 h and 30 min at 300 mA, and then blocked in 5% non-fat milk in Tris-buffered saline-Tween (TBS-T) solution for 1 h, at RT. Membranes were incubated overnight at 4 °C with primary antibodies: anti-STAT3, mouse monoclonal (BioLegend, Cat no 678001), anti-phospho-STAT3 (Tyr705), mouse monoclonal (BioLegend, Cat no 651001), anti-GAPDH, mouse monoclonal (BioLegend, Cat no 649201), anti-Ki-67, mouse monoclonal (BioLegend, Cat no 350501), anti-cleaved caspase 3, rabbit monoclonal (Cell Signaling, Cat no 9661S), anti-Mcl-1, rabbit monoclonal (Cell Signaling, Cat no 5453T), anti-alpha Tubulin, rabbit polyclonal (Abcam, Cat no ab15246) and anti-Pim-1, rabbit monoclonal (Cell Signaling, Cat no 3247T). Antibody dilutions were 1:1000, except for anti-α Tubulin at 1:8000, following manufacturers’ instructions. After three washes with TBS-T (5 min each), membranes were incubated with the horseradish peroxidase (HRP)-conjugated secondary antibodies: HRP-goat anti-mouse polyclonal antibody (BioLegend, Cat no 405306) and HRP-mouse anti-rabbit polyclonal antibody (Invitrogen, Cat no 61–6520), at a dilution of 1:4000 each, for 1 h, at RT. All the antibodies were diluted in the blocking solution. Subsequently, membranes were washed three times with TBS-T (10 min each), then developed with either Immobilon^®^ Crescendo Western HRP substrate (Merck, Cat no WBLUR0100), or SuperSignal™ West Femto Maximum Sensitivity Substrate (Thermo Fisher, Cat no 34094). Protein bands were visualized using the ChemiDoc™ MP Imaging System (Bio-Rad), and densitometry analysis was performed with ImageJ software (National Institutes of Health).

### 2.12 Data analysis and statistics

All experiments were performed independently in triplicate (*n* = 3), with three biological replicates throughout the study. Data are presented as means ± SD from technical replicates or ±SEM from three independent experiments. Statistical significance was assessed using one-tailed unpaired *t*-test and one-way ANOVA, using GraphPad Prism v9.3.0 (Dotmatics). A *p*-value of <0.05 was considered statistically significant. Inhibitory dose-response curves, IC_50_ calculations, and quantitation graphs were obtained using GraphPad Prism software. The 3D structure prediction was obtained using the AlphaFold 3 Server (Abramson et al., 2024).

## 3 Results

### 3.1 Design of minicircles and the principle of circularization

**FIGURE 1:**
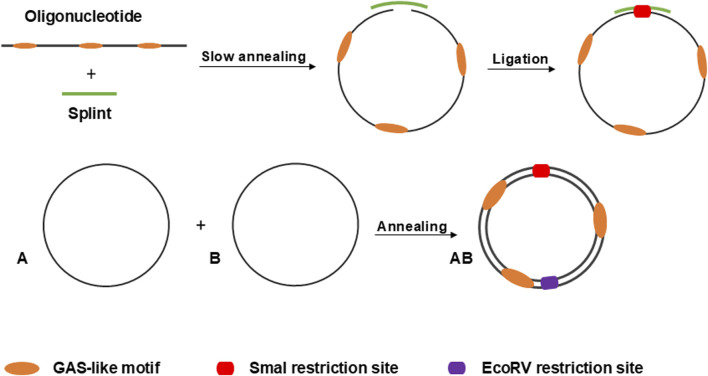
Schematic overview of the process for circularizing oligonucleotides via the splint-assisted enzymatic ligation method. The annealing of two complementary single-stranded circles results in the formation of mcDNA molecules. The SmaI restriction site is used to verify successful circularization, while EcoRV digestion confirms the double-stranded status of the minicircles.

In this study, we utilized linear 5′-phosphorylated DNA molecules of 95 nucleotides in length, each containing three equidistant GAS-like sequences. First, two single-stranded complementary oligonucleotides were circularized separately using the “Double-helical complex splint-assisted enzymatic cyclization of oligonucleotides with T4 DNA ligase” method (Diegelman and Kool, 2000). This process employs a splint–a specific short single-stranded DNA molecule complementary to the ends of the oligonucleotide. During the reaction, the ends of the oligonucleotide are slowly brought together through annealing with the splint, followed by a very slow specific ligation. After obtaining the single-stranded circles, they are annealed based on their complementarity to form the final double-stranded circular DNA molecule, the anti-STAT3 minicircle DNA (anti-STAT3 mcDNA). The negative control minicircle is similarly obtained, but contains mutated GAS-like sequences (mock mcDNA). Figure 1 provides a schematic overview of the process used to obtain the minicircles.

### 3.2 Development and validation of DNA minicircles

**FIGURE 2:**
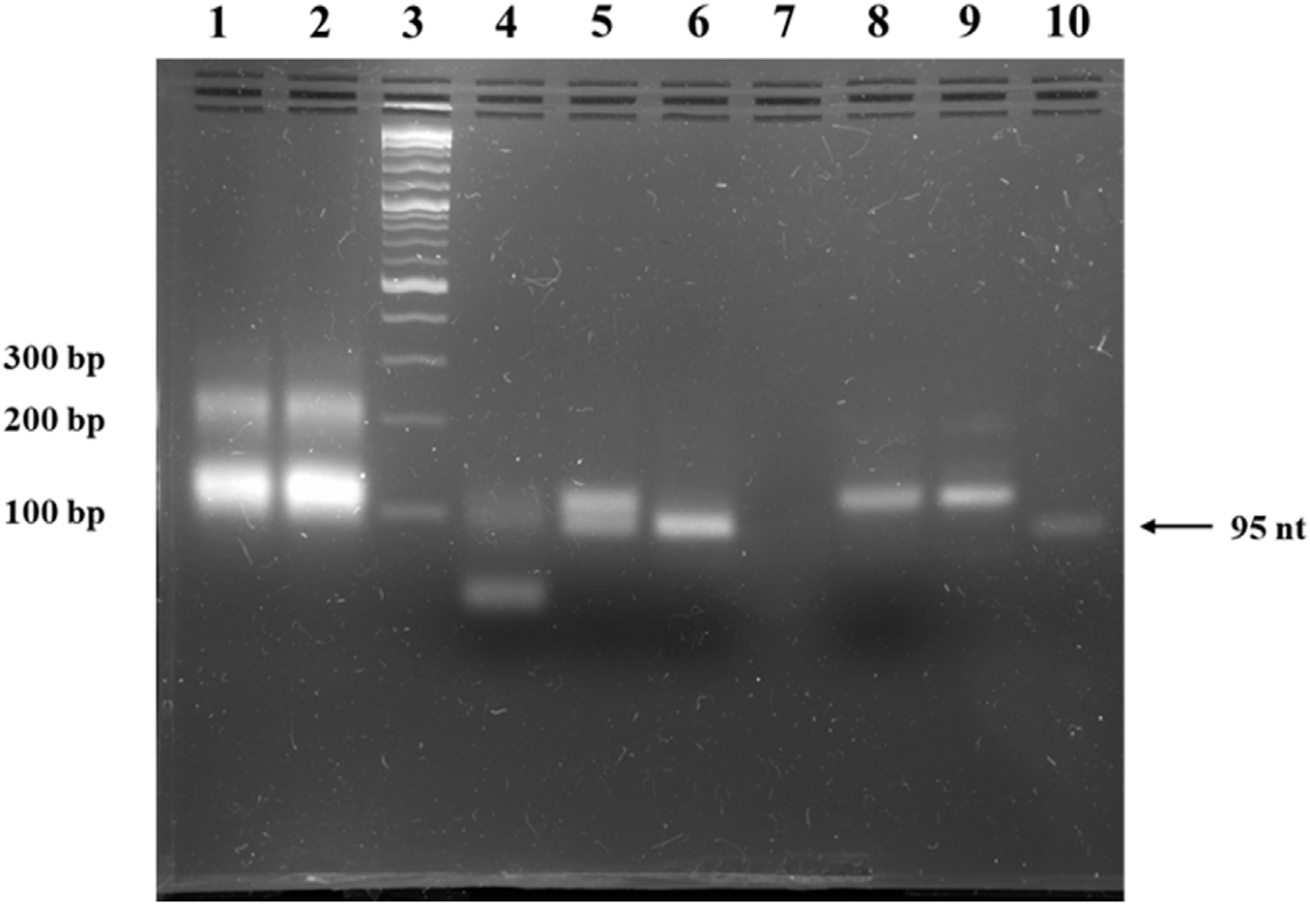
Agarose gel electrophoresis of anti-STAT3 mcDNA. Lane 1: ligation product of single-stranded minicircle A; Lane 2: ligation product of the complementary single-stranded minicircle B; Lane 3: Quick-Load^®^ 1 kb Plus DNA Ladder; Lane 4: purified, annealed double-stranded minicircle digested with both SmaI and EcoRV; Lane 5: purified, annealed double-stranded minicircle digested with SmaI alone; Lane 6: purified, annealed double-stranded minicircle digested with EcoRV alone; Lane 7: empty lane; Lane 8: negative control of purified, annealed double-stranded minicircle incubated without restriction enzymes; Lane 9: purified, annealed double-stranded minicircle DNA; Lane 10: linear oligonucleotide precursor for the single-stranded minicircle A.

To obtain the double-stranded anti-STAT3 and mock minicircles, we circularized the corresponding oligonucleotides using specific splints, establishing a unique SmaI restriction site at the ligation point within the newly formed double-stranded region (oligonucleotide plus splint). Upon annealing, the complementary single-stranded circles form a final molecule where, on the opposite side of the SmaI site, a unique EcoRV restriction site is generated. Thus, the SmaI site confirms circularization, while the EcoRV site verifies the complete annealing of the two complementary single-stranded circles. As shown in Figure 2, the final molecule adopts a double-stranded circularized form. After digestion with both SmaI and EcoRV, the major product migrates considerably lower than the simple 95-nucleotide oligo. SmaI digestion results in two bands: the higher one corresponds to the intact double-stranded circular molecule and the lower one (∼100 bp) indicates a linear fragment, with only partial digestion. EcoRV digestion produces a single band around 100 bp, different from the final circular product, similar to the lower band observed after digestion with SmaI. As a technical negative control, we used purified, annealed double-stranded minicircle incubated without restriction enzymes. This control confirmed the circularity and double-stranded state of the obtained molecules based on the enzymatic digestion results.

The result of the enzymatic circularization and ligation reaction present in lanes 1 and 2 of Figure 2 reveals extra bands at ∼200 bp. To verify the identity of these bands, we isolated the complementary ∼200-nucleotide strands from the agarose gel, annealed them and then performed a restriction test on the resulting double-stranded DNA similar to the one utilized in Figure 2. Thus, the *SmaI* and *EcoRV* enzymatic digestion led to an identical band pattern to the pattern observed in the case of the minicircles (Supplementary Figure S1).

### 3.3 Anti-STAT3 mcDNA specifically binds STAT3 protein

**FIGURE 3:**
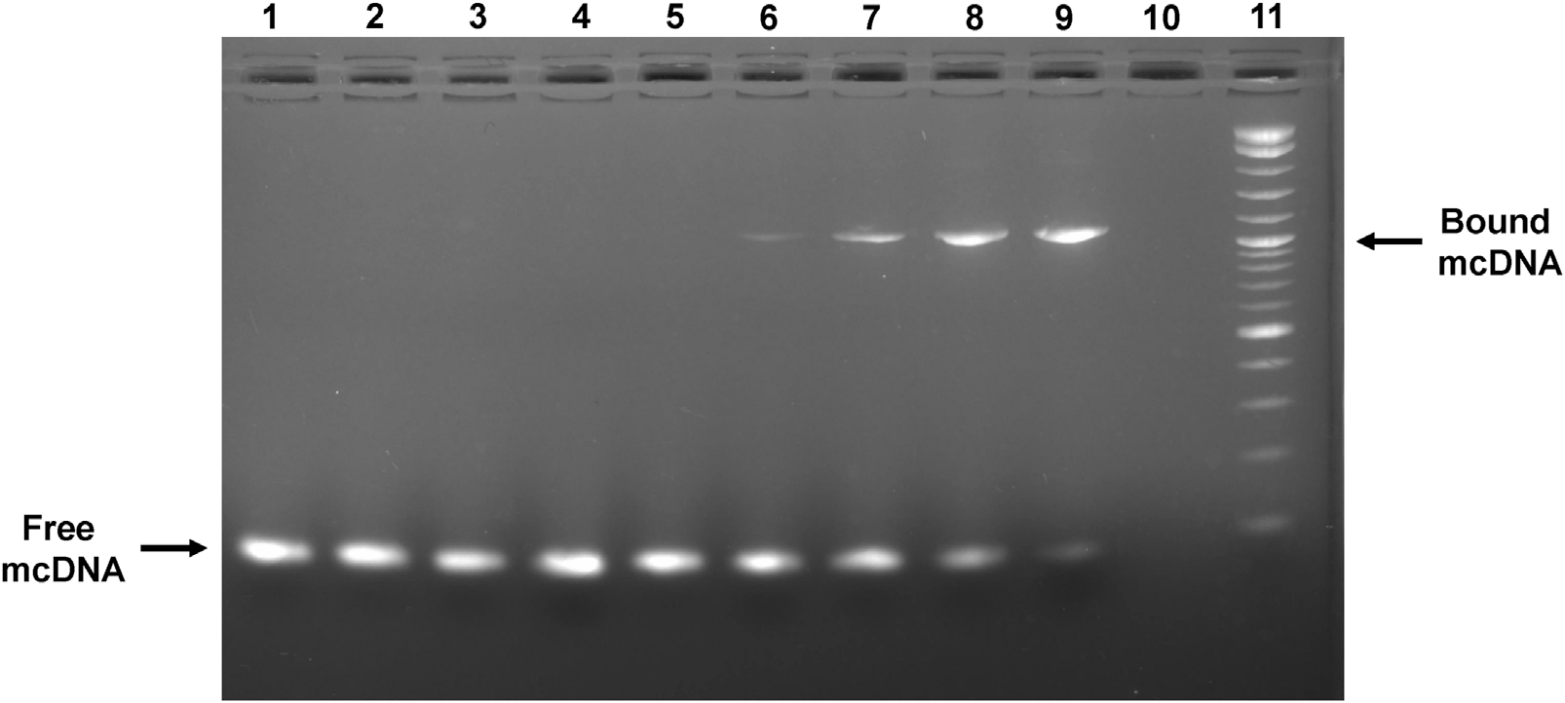
Agarose gel showing the interaction between the anti-STAT3 mcDNA and STAT3 protein in an EMSA assay. Binding was performed in varying minicircle:STAT3 protein molar ratios. Lane 1: mock mcDNA, without protein; Lane 2: mock mcDNA:STAT3 in a ratio of 1:3; Lane 3: mock mcDNA:STAT3 in a ratio of 1:4; Lane 4: anti-STAT3 mcDNA, without protein; Lane 5: anti-STAT3 mcDNA:STAT3 in a ratio of 1:0.75; Lane 6: anti-STAT3 mcDNA:STAT3 in a ratio of 1:1.5; Lane 7: anti-STAT3 mcDNA:STAT3 in a ratio of 1:2; Lane 8: anti-STAT3 mcDNA:STAT3 in a ratio of 1:3; Lane 9: anti-STAT3 mcDNA:STAT3 in a ratio of 1:4; Lane 10: empty; Lane 11: Quick-Load^®^ 1 kb Plus DNA Ladder. The arrows indicate unbound minicircle DNA and anti-STAT3 mcDNA bound to STAT3, respectively.

To assess the capability of the anti-STAT3 mcDNA to be recognized by and interact with the STAT3 protein, we performed an EMSA assay utilizing various molar ratios of DNA:protein. Figure 3 shows a mobility shift in the migration of anti-STAT3 mcDNA bound to STAT3 that gradually increases with protein quantity. The shift takes place in the range 1:1.5–1:4 from the tested molar ratios, which confirms that anti-STAT3 mcDNA binds to STAT3. In contrast, mock mcDNA did not show a migration shift in any of the tested conditions, similar to the technical negative control of DNA without protein. Taken together, these results show that anti-STAT3 mcDNA specifically interacts with the STAT3 protein.

### 3.4 Anti-STAT3 mcDNA reduces the viability of SKOV3 ovarian cancer cells

To evaluate the transfectability of the SKOV3 ovarian cancer cell line and to identify the transfection reagent with the highest transfection efficiency, we transfected SKOV3 cells with a GFP-expressing plasmid using various DNA-to-reagent ratios for Lipofectamine 3000 and PEI, respectively. Supplementary Figure S2 presents the flow cytometry results obtained 48 h post-transfection. Supplementary Figure S2A shows the gating strategy used to identify the GFP-positive cells in Cytobank. As shown in Supplementary Figure S2B, Lipofectamine 3000 was the most effective transfection reagent for SKOV3 cells, with the optimal DNA: Lipofectamine ratio of 1:1, achieving over 50% transfection efficiency, while a ratio of 1:2 resulted in approximately 34% transfection efficiency. All results were compared with negative controls, which included Lipofectamine-only, PEI-only transfections, and untreated cells. Based on these results, all subsequent transfections performed in this study employed the optimal 1:1 DNA: Lipofectamine ratio.

**FIGURE 4:**
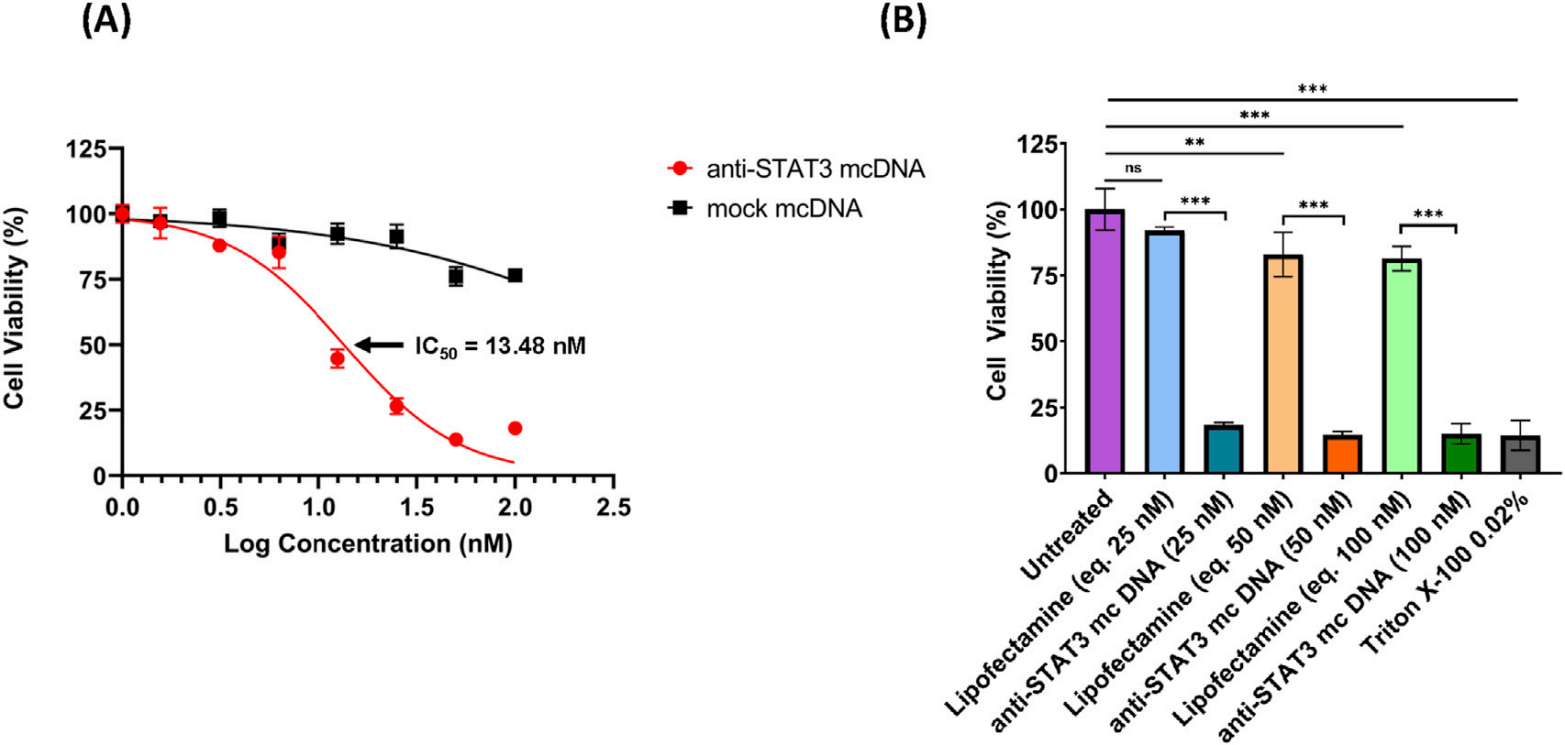
The inhibitory effect of anti-STAT3 mcDNA on SKOV3 ovarian carcinoma cells, assessed by the MTS assay. **(A)** Dose-response curves showing the effect of anti-STAT3 mcDNA and mock mcDNA transfected into SKOV3 cells. Data points represent the mean percentage cell viability normalized to Lipofectamine-only controls at treatment concentrations: 0 nM, 1.56 nM, 3.12 nM, 6.25 nM, 12.5 nM, 25 nM, 50 nM and 100 nM. **(B)** Toxicity of Lipofectamine 3000 alone on SKOV3 cells at equivalent DNA concentrations. Statistical significance was determined by one-way ANOVA. ns–not significant (*p* ≥ 0.05); *p < 0.05; **p < 0.01; ***p < 0.001. Error bars indicate ±SD. Data show the results from a representative experiment out of three performed (*n* = 3).

As we observed a high transfection rate for SKOV3 cells utilizing Lipofectamine 3000, we proceeded to assess the effect of anti-STAT3 mcDNA on cell viability. SKOV3 cells were transfected with anti-STAT3 minicircle or mock minicircle at the concentrations: 1.56 nM, 3.12 nM, 6.25 nM, 12.5 nM, 25 nM, 50 nM and 100 nM in technical triplicate. Lipofectamine-only transfection mixes corresponding to 25 nM, 50 nM and 100 nM compound were used as the technical negative controls. The technical positive control was a 15-min treatment with Triton X-100 0.02%. After 48 h, cell viability was assessed using the MTS assay. As shown in Figure 4A, the IC_50_ value of the anti-STAT3 compound was 13.48 nM, with cell viability decreasing in a dose-dependent manner compared to Lipofectamine-only-treated cells. In contrast, the mock mcDNA did not significantly influence cell viability. Notably, we observed a statistically significant toxic effect in Lipofectamine-only-treated cells for the concentrations equivalent to 50 nM and 100 nM DNA, compared to untreated cells, but not at 25 nM (Figure 4B). These findings suggest that at higher Lipofectamine concentrations, the reagent`s toxicity could contribute to the observed effects. However, at 25 nM, Lipofectamine exhibits no toxic effect, indicating that the reduction in viability at the IC_50_ was entirely due to the anti-STAT3 mcDNA treatment. Moreover, we observed a significant difference in cell viability between cells treated with the anti-STAT3 mcDNA and those treated with Lipofectamine 3000 alone. Together with the negligible effect of mock mcDNA, these results confirm the specific inhibitory activity of anti-STAT3 mcDNA.

### 3.5 Inhibition of STAT3 activity by anti-STAT3 mcDNA induces apoptosis and necrosis in representative ovarian cancer cells

**FIGURE 5:**
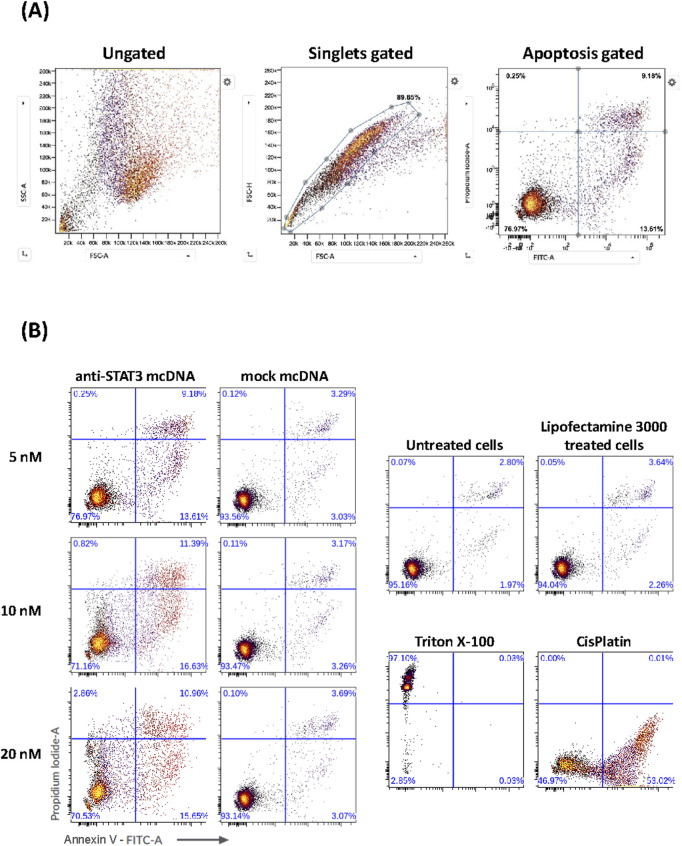
Effect of anti-STAT3 mcDNA on apoptosis and necrosis in SKOV3 OC cells. **(A)** Gating strategy used to identify apoptotic cells by flow cytometry. **(B)** Percentage of apoptotic and necrotic cells following treatment with various concentrations of anti-STAT3 mcDNA compared to the mock control. The lower right quadrant shows apoptotic cells (Annexin V^+^, PI^−^) and the upper right quadrant shows necrotic cells (Annexin V^+^, PI^+^). Data show the results from a representative experiment out of three performed (*n* = 3).

Given the very promising and specific SKOV3 viability reduction that we observed for anti-STAT3 mcDNA in the MTS assay, we proceeded to evaluate the underlying effects of our compound on apoptosis and necrosis in these representative ovarian cancer cells. SKOV3 cells were transfected with three concentrations of anti-STAT3 mcDNA (5 nM, 10 nM and 20 nM) and compared to the mock control. Forty-eight hours post-transfection with anti-STAT3 mcDNA, flow cytometry revealed a dose-dependent increase in apoptosis and necrosis (Figure 5). The technical positive controls were Cisplatin (apoptosis) and Triton X-100 (necrosis) (Figure 5B). Figure 5A shows the gating strategy to identify apoptotic cells. Treatment with anti-STAT3 mcDNA increased the percentage of apoptotic (Annexin V^+^, PI^−^) and necrotic (Annexin V^+^, PI^+^) cells at all three tested concentrations, whereas mock mcDNA (negative control for specificity) had almost no effect on cell survival, similar to cells treated with Lipofectamine alone (technical negative control), regardless of concentration (Figure 5B). Even at 5 nM, anti-STAT3 mcDNA markedly reduced cell viability, with 76.97% cells remaining viable, while 9.18% were necrotic and 13.61% tested apoptotic.

Increasing the concentration to 10 nM further increased apoptosis and necrosis, with effects plateauing at higher concentrations. These findings demonstrate that anti-STAT3 mcDNA promotes apoptosis and necrosis in SKOV3 cells in a dose-dependent manner within the 0–10 nM range.

### 3.6 Anti-STAT3 mcDNA treatment decreases proliferation of SKOV3

**FIGURE 6:**
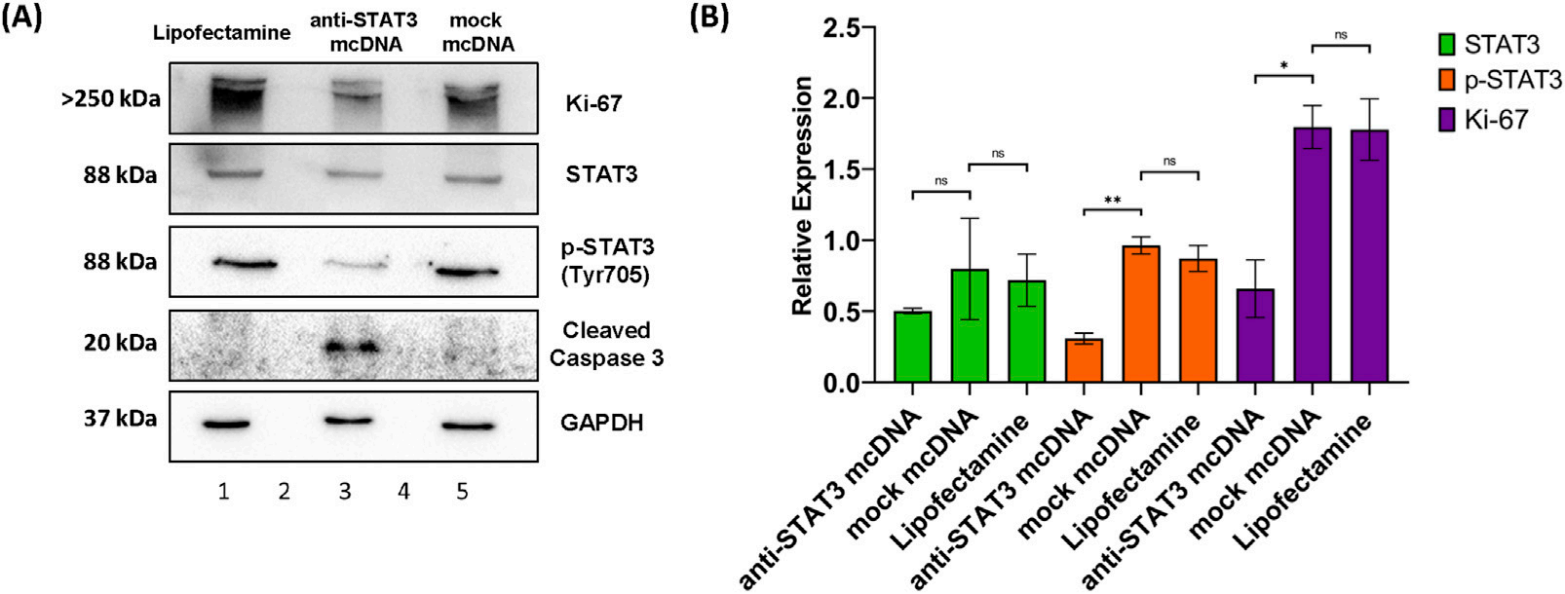
Western blot analysis of SKOV3 cell lysates. **(A)** Protein expression patterns of Ki-67, STAT3, p-STAT3 (Tyr705), cleaved caspase-3 and GAPDH in SKOV3 cells treated with Lipofectamine 3000 alone (lane 1), anti-STAT3 mcDNA (lane 3) and mock mcDNA (lane 5) at a concentration of 10 nM. Lanes 2 and 4 are empty. The approximate molecular weight of each band is indicated on the left. **(B)** Quantification of STAT3, p-STAT3 and Ki-67 levels, normalized to GAPDH as a loading control, across treatments. Cleaved caspase-3 is shown as present or absent due to its low abundance. One-tailed unpaired *t*-test determined statistical significance. ns–not significant (*p* ≥ 0.05); *p < 0.05; **p < 0.01. Error bars represent ±SEM; *n* = 3.

To further evaluate the effects of anti-STAT3 mcDNA on SKOV3 OC cells, we determined the levels of STAT3, phosphoTyr705-STAT3 (p-STAT3), Ki-67 and cleaved caspase-3 by Western blot analysis of total cell lysates after treatment with 10 nM of anti-STAT3 mcDNA and mock mcDNA (experimental negative control). The STAT3 protein expression in cells treated with anti-STAT3 mcDNA was slightly lower than in cells treated with Lipofectamine alone (technical negative control), or mock mcDNA (Figure 6); however, the difference was not statistically significant. The levels of p-STAT3 we observed in Lipofectamine only and mock mcDNA-treated samples indicate the presence of active STAT3. However, p-STAT3 was significantly lower in cells treated with anti-STAT3 mcDNA, compared to the controls, indicating impaired signaling in the JAK/STAT3 pathway. In addition, Ki-67 levels were over two-fold lower in cells treated with the anti-STAT3 mcDNA compared to control, achieving statistical significance and indicating suppressed proliferation. The levels of Ki-67 in Lipofectamine and mock-treated cells were comparable. In line with the flow cytometry results, which indicated induction of apoptosis, cleaved caspase-3 was detected solely in cells treated with the anti-STAT3 construct. These findings therefore confirm that anti-STAT3 mcDNA not only induces apoptosis and necrosis, but also inhibits STAT3 signaling, thus decreasing cell proliferation in SKOV3 cells.

### 3.7 Inhibition of STAT3 reduces anti-apoptotic and pro-survival gene expression

**FIGURE 7:**
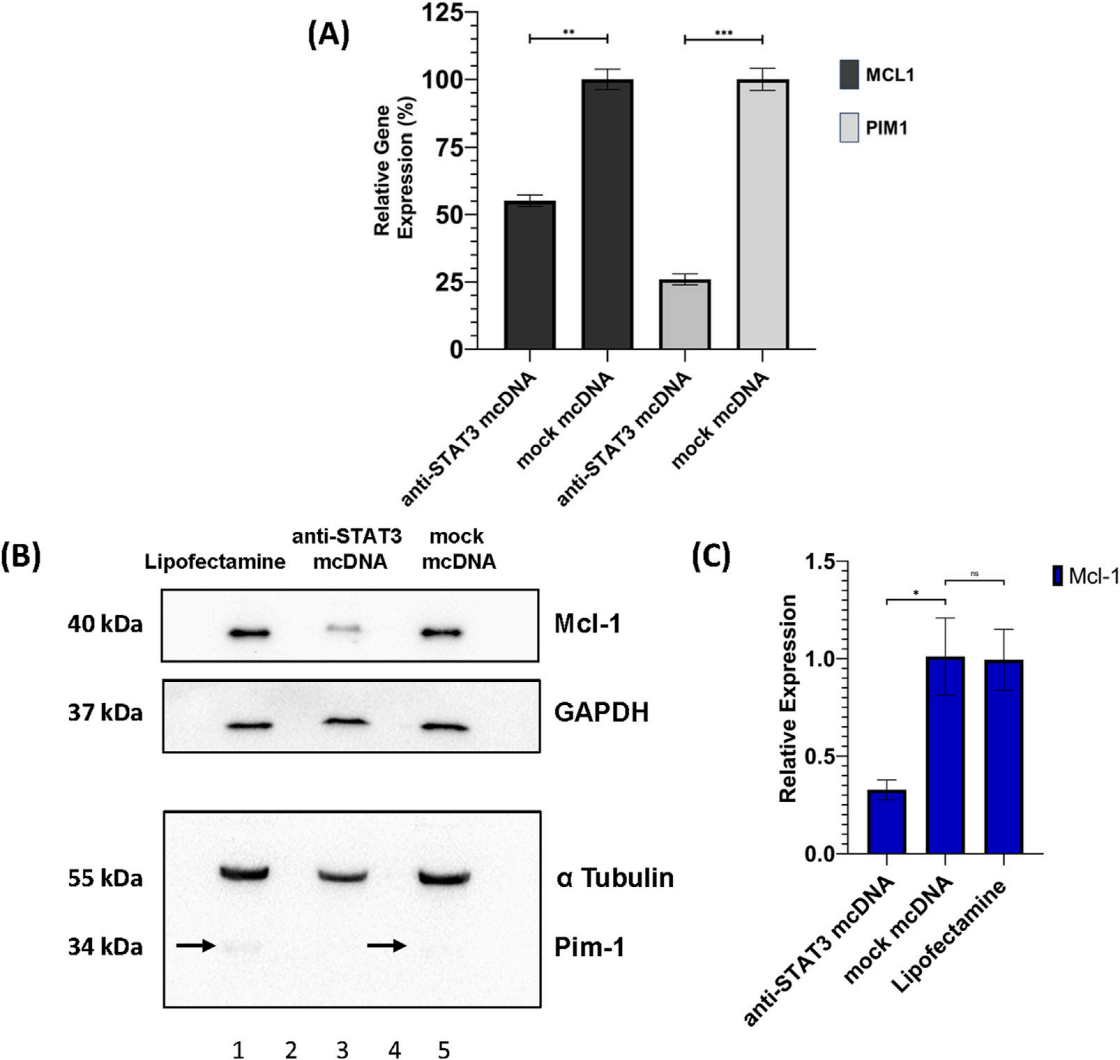
Effects of anti-STAT3 mcDNA treatment on downstream targets. **(A)** Quantification of the Relative Gene Expression through RT-qPCR of the *MCL1* and *PIM1* genes, measured in anti-STAT3 mcDNA and mock mcDNA treatment conditions (treatment concentration of 10 nM), normalized to *GAPDH* gene expression. One-tailed unpaired *t*-test was used to determine statistical significance. Error bars represent ±SD. Data show the results from a representative experiment out of three performed (*n* = 3). Western blot analysis of SKOV3 cell lysates. **(B)** Expression patterns of Mcl-1, Pim-1, GAPDH and α-Tubulin in the SKOV3 cells treated with Lipofectamine 3000 alone (lane 1), anti-STAT3 mcDNA (lane 3) and mock mcDNA (lane 5) at a concentration of 10 nM. Lanes 2 and 4 are empty. On the left side, the approximate molecular weights (kDa) of each band are indicated. The black arrows mark Pim-1 protein bands. **(C)** Quantification of Mcl-1 protein levels normalized to GAPDH across treatment conditions. Pim-1 was only observed as present or absent due to its extremely low abundance. Statistical significance was assessed using a one-tailed unpaired *t*-test. ns–not significant (*p* ≥ 0.05); *p < 0.05; **p < 0.01; ***p < 0.001. Error bars represent ±SEM; *n* = 3.

To assess the impact of STAT3 inhibition on the expression of downstream target genes as a possible cause for the treated cell phenotypes observed in MTS and flow cytometry, we performed RT-qPCR analysis of *MCL1* and *PIM1* in SKOV3 cells treated with 10 nM anti-STAT3 mcDNA, or mock mcDNA, respectively. Experiments were performed employing technical duplicates. As shown in Figure 7A, both genes were significantly downregulated in anti-STAT3 mcDNA-treated cells compared to mock mcDNA (negative control). Particularly, *MCL1* expression decreased by approximately 50% (*p* < 0.01), while *PIM1* expression was reduced by about 75% (*p* < 0.001). These results therefore indicate that inhibition of STAT3 activity leads to downregulation of key anti-apoptotic and pro-survival genes in the tested representative OC cells.

Furthermore, we also assessed the protein levels of Mcl-1 and Pim-1 by Western blot in SKOV3 cells. Mcl-1 protein expression was significantly reduced (over 2-fold) in anti-STAT3 mcDNA-treated cells, compared to mock mcDNA (experimental negative control) (Figures 7B,C), consistent with the mRNA data. Mock mcDNA-treated cells displayed Mcl-1 levels comparable to those treated with Lipofectamine alone (technical negative control). Due to low abundance of Pim-1 protein, quantification was not feasible; the bands indicated by black arrows were barely detectable and no band was observed in the anti-STAT3 mcDNA-treated samples.

These findings, together with the RT-qPCR results, suggest that anti-STAT3 mcDNA effectively reduces the expression of STAT3 target genes, contributing to decreased survival and proliferation of SKOV3 ovarian cancer cells.

## 4 Discussion

STAT3 is an essential transcription factor involved in regulating numerous genes controlling critical cellular processes, including cell growth, survival, proliferation, immune response and stemness (Levy and Lee, 2002). In cancer, aberrantly active STAT3 drives oncogenic programs, promoting cell-cycle progression, uncontrolled proliferation, resistance to apoptosis, and tumor neoangiogenesis (Hu et al., 2024).

Several strategies have been developed over time to inhibit STAT3 expression or activity. One such strategy is the use of small molecules that inhibit STAT3 signaling through binding its SH2 or DBD domain, like STATTIC or Napabucasin. However, in the clinical setting, they displayed non-specific activity and high toxicity, or their effects were not highly significant, as the patient sample size was too low, which drastically reduce their relevance as therapeutics (Schust et al., 2006; Shitara et al., 2019). Another class of inhibitors that are able to impair the STAT3 signaling pathway are JAK2 inhibitors. These have proven effects on various cancer types, yet they are also plagued by reduced specificity, which causes serious side effects. Additionally, a difficult and well-known problem with targeting JAK2 is that the cancer cells treated with this type of inhibitors frequently develop resistance to them. Such is the case with the prominent JAK2 inhibitor Ruxolitinib, which proved to be temporarily effective in early stages of clinical trials, yet later it induced resistance mechanisms and various side effects like thrombocytopenia and reactivation of opportunistic infections (Nair et al., 2023). A newer direction that was developed for STAT3 inhibition makes use of a diverse array of nucleic acids. siRNA molecules have been proven to be effective, but with the caveat of stability issues and off-target effects (Sun et al., 2015; Han et al., 2013). As a significant step forward, decoy oligodeoxynucleotides (ODN-decoy) offer numerous advantages, such as high affinity for their target, accessibility, specificity, and demonstrated effectiveness (Crinelli et al., 2002). A linear 15 bp ODN-decoy has been tested in various types of cancer models, including ovarian cancer (OC) (Liu et al., 2014). Despite showing promise, this linear decoy failed to exert significant effects in xenograft models, likely due to its thermosensitivity and fast degradation by nucleases in the serum (Sen et al., 2012). To overcome these limitations, a cyclic decoy molecule was developed using hexaethylene glycol linkers, which proved effective in head and neck cancer xenograft models (Sen et al., 2014).

This study highlights the potential of targeting STAT3 activity in SKOV3 OC cells using a novel double-stranded DNA circular molecule designed to specifically bind the DBD of STAT3. The anti-STAT3 minicircle contains three equally spaced GAS-like motifs, increasing the likelihood of interaction with STAT3 and achieving targeted inhibition. The anti-STAT3 mcDNA we developed may offer improved target selectivity for inhibiting the STAT3 signaling pathway with minimal off-target effects compared to small-molecule or JAK2 inhibitors (Schust et al., 2006; Shitara et al., 2019; Nair et al., 2023). This minicircle, given its structure, is potentially more stable *in vitro* and *in vivo* than siRNA molecules or linear ODN decoys (Sun et al., 2015; Han et al., 2013; Casas et al., 2022) and with much simpler chemistry than the chemically circularized decoy molecules previously reported (Sen et al., 2014).

We selected the GAS-like sequence 5′ TTCCCGTAA 3′ due to its status as a consensus sequence with high affinity for STAT3 (Yang et al., 2003). In fact, a 3D structure prediction of a STAT3 dimer, together with a GAS-like region from the anti-STAT3 mcDNA molecule, also suggests an interaction between the two (Supplementary Figure S3). To verify the efficacy of our construct, we successfully obtained and validated both the anti-STAT3 mcDNA and the corresponding mock control, which lacks functional GAS-like motifs. Our observations on gel mobility revealed that short oligonucleotides of similar length exhibit predictable migration patterns: linear single-stranded DNA migrates the fastest, followed by circular single-stranded DNA, which migrates similarly to the circular double-stranded DNA. This observation is important for future purification strategies. The migration differences are most likely due to the small size of the precursor oligonucleotides (95 nucleotides), used to form the minicircles. Given their length, they are less prone to forming secondary or supercoiled structures. The similar restriction pattern between the lower and higher bands in Figure 2 is expected to occur in the case of multimer formation. Thus, the ∼200 bp bands analyzed in Supplementary Figure S1 most likely represent circular dimers formed as secondary byproducts of the protocol we employed, given their length. They are the minority products, yet they are expected to form in this technique, as described in the original protocol (Diegelman and Kool, 2000).

A valuable study by Casas et al. (2022) reported STAT3-targeting DNA minicircles with convincing activity in triple-negative breast cancer cells. Our study is conceptually related but differs in methodology: we generated minicircles by enzymatic ligation using complementary splints, whereas Casas et al. employed overlapped nicked duplexes. In addition, we validated circularity and purity through specific restriction assays, which we consider essential for downstream cellular and *in vivo* applications. Both studies demonstrate direct interaction of minicircles with STAT3 (Casas et al. by pull-down assays, our work by EMSA), as well as comparable cell inhibitory effects. Taken together, these findings support the robustness of STAT3 inhibition by minicircle DNA across different cellular models.

The SKOV3 ovarian cancer cell line, characterized by the fifth highest level of STAT3 expression (The Human Protein Atlas, 2025), was chosen as our model. We first evaluated its transfectability and transfection efficiency. Flow cytometry on GFP-transfected cells indicated that Lipofectamine 3000 achieved the best transfection efficiency, leading to an ∼50% transfection rate, when using a 1:1 DNA: Lipofectamine ratio. In agreement with previous studies, toxicity increases at higher Lipofectamine doses (Sun et al., 2008), explaining the lower transfection rate at a 1:2 ratio.

Our functional assays revealed that the anti-STAT3 mcDNA, 48 h post-transfection, has a promising IC_50_ of 13.48 nM - a low nanomolar concentration comparable or superior to previously reported decoy molecules used in head and neck cancer models (Sen et al., 2012). The IC_50_ we obtained is also supported by the work of Casas et al. (Casas et al., 2022), where a similar potency of the DNA minicircle was obtained against MDA-MB-231 breast cancer cells. The increased structural stability conferred by the minicircle architecture likely underpins this efficiency, providing extended half-life and higher resistance to serum and intracellular nucleases, compared to a linear inhibitor, all valuable properties that were previously demonstrated in syngeneic triple-negative breast cancer xenografted mice (Casas et al., 2022).

Flow cytometry demonstrated that treatment with anti-STAT3 mcDNA induced apoptosis and necrosis across all tested concentrations (5, 10, and 20 nM), effects that were absent in cells treated with mock mcDNA. These results demonstrated the specificity of minicircles in targeting the STAT3 protein thanks to the presence of GAS-like motifs. The similar effect between 10 nM and 20 nM anti-STAT3 mcDNA treatment, in the absence of any toxic effect from Lipofectamine, could be explained by the saturation of cells upon transfection. Our results are also supported by a previous study in which a very similar apoptosis and necrosis effect was obtained, utilizing DNA minicircles (Casas et al., 2022).

Western blot further confirmed the obtained results: cleaved caspase-3, a hallmark of both the intrinsic and extrinsic apoptosis pathways, was detected only in anti-STAT3-treated cells, while the levels of proliferation marker Ki-67 were significantly reduced, compared to mock-treated cells, indicating suppressed cell cycle progression (Eskandari and Eaves, 2022; Sun and Kaufman, 2018). Although a tendency toward decreased STAT3 protein levels was observed in the anti-STAT3 mcDNA-treated group, statistical significance was not achieved. This trend may reflect autoregulatory feedback, as STAT3 is known to regulate its own expression (Ichiba et al., 1998). Strengthening these findings, the levels of p-STAT3 observed in the case of treatment with our compound are significantly reduced, probably also due to this autoregulatory feedback. Another cause for this lower signal of p-STAT3 may be a downstream inhibition of the pathway responsible for STAT3 phosphorylation. Notably, RT-qPCR and correlated Western blot analyses demonstrated that treatment with anti-STAT3 mcDNA significantly downregulates key STAT3 targets, such as anti-apoptotic and pro-survival genes *MCL1* (both at the mRNA and protein levels) and *PIM1* (at the mRNA level), further supporting the biological efficacy of our construct. This downregulation of the *MCL1* and *PIM1* genes in the case of anti-STAT3 mcDNA treatment seems to be a direct consequence of the inhibition of STAT3 activity.

This work provides an initial proof-of-concept for a novel circular DNA-based inhibitor that effectively impairs cell growth and survival *in vitro* in the SKOV3 model for ovarian cancer via specific modulation of STAT3 activity. The main limitations are the use of a single cell line, the absence of *in vivo* validation, and the lack of large-scale production methods (e.g., HPLC purification), all essential for comprehensive preclinical evaluation.

To move towards translational studies, delivery strategies such as solid lipid nanoparticles (SLN) can be employed. The advantages of using SLNs over other lipid emulsions, such as liposomes, as carriers of the active minicircles are: protection and stability for the encapsulated drug, the ability to control the drug release *in vivo*, minimal cytotoxicity and high safety profiles (Akanda et al., 2023). All these beneficial properties can be coupled with effective targeting methods against ovarian cancer and other types of malignancies, utilizing highly specific biomarkers present on the surface of tumor cells, such as B7-H3 (He et al., 2025). To this end, various antibodies or affibodies raised against these biomarkers can be linked to the SLN through various methods (Akanda et al., 2023). This is the main strategy we envision for a future safe personalized ovarian cancer therapy utilizing the anti-STAT3 mcDNA developed in this study.

In a future study, we plan to test the compound on additional ovarian cancer cell lines with diverse genetic backgrounds, followed by evaluation of its stability and efficacy in xenograft mouse models. These investigations will be essential to assess translational potential and to explore the feasibility of advancing this approach toward clinical application in ovarian cancer.

In conclusion, the unique structural features of our anti-STAT3 mcDNA - including the presence of three GAS-like motifs, its circular and double-stranded configuration and its chemical simplicity - confer enhanced specificity, increased probability of interaction with the target, high stability, increased nuclease resistance and cost-effective production, including via bacterial cloning. These attributes position anti-STAT3 mcDNA as a promising future therapeutic candidate for downregulation of STAT3 activity. Our findings support further development toward preclinical validation and, ultimately, clinical application of this technology, not only in ovarian cancer but also in other STAT3-driven malignancies. Given these strengths, anti-STAT3 mcDNA may represent a potentially effective therapeutic approach for modulating STAT3 activity in cancer.

## Data Availability

The original contributions presented in the study are included in the article/Supplementary Material, further inquiries can be directed to the corresponding authors.

## Abbreviations

STAT3, Signal Transducer and Activator of Transcription 3; JAK, Janus kinase; mcDNA, minicircle DNA; OC, ovarian cancer; PEI, polyethylenimine.
