# Supplementary material for: Breaking the cancer code: a novel DNA minicircle to disable STAT3 in ovarian cancer cells SKOV3
Source: Front Pharmacol. 2025 Sep 15;16:1673427. doi: 10.3389/fphar.2025.1673427 (PMC12477429; doi:10.3389/fphar.2025.1673427)
Supplement: Supplementary file 1 [file DataSheet1.docx]

Supplementary Material

# Supplementary Figures and Tables

##
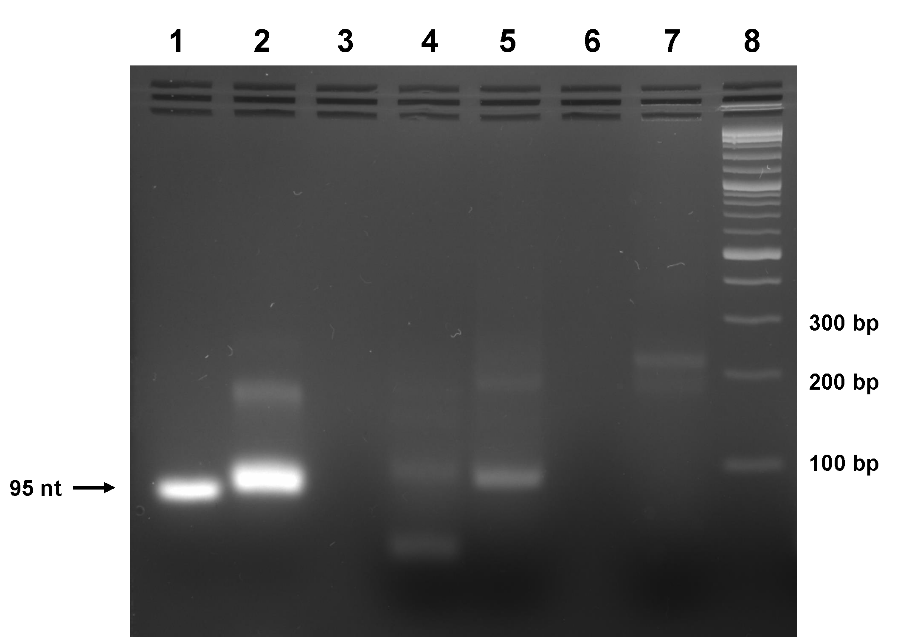
Supplementary Figures

**Supplementary Figure S1**. Agarose gel for the restriction digest of the purified ~200 bp double-stranded band. Lane 1: linear oligonucleotide precursor for the single-stranded minicircle; Lane 2: ligation product of one of the two complementary single-stranded minicircles; Lane 3: empty; Lane 4: purified, annealed double-stranded ~200 bp product digested with both SmaI and EcoRV; Lane 5: purified, annealed double-stranded ~200 bp product digested with SmaI alone; Lane 6: empty; Lane 7: negative control of purified, annealed double-stranded ~200 bp product incubated without restriction enzymes; Lane 8: Quick-Load® 1 kb Plus DNA Ladder.


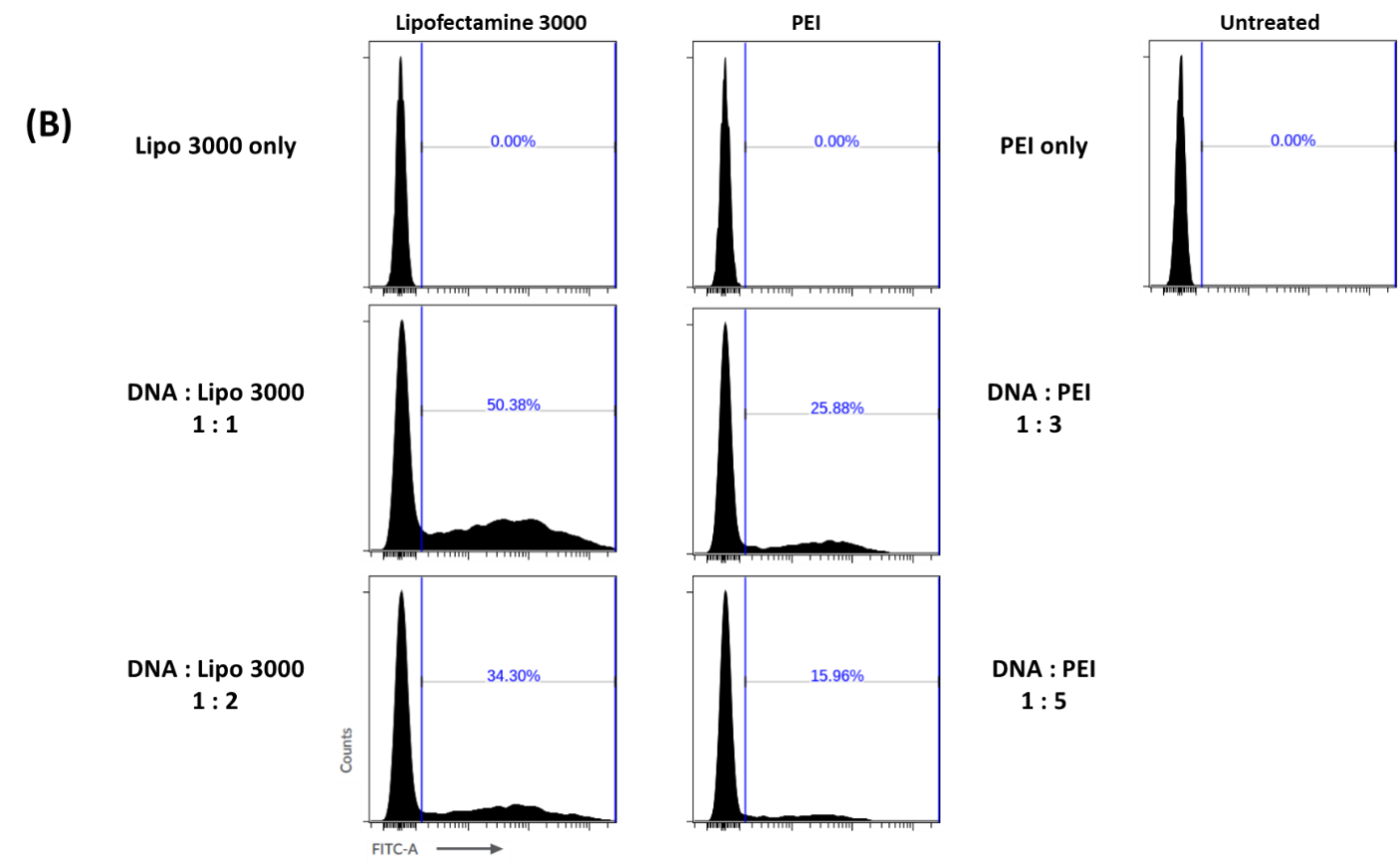

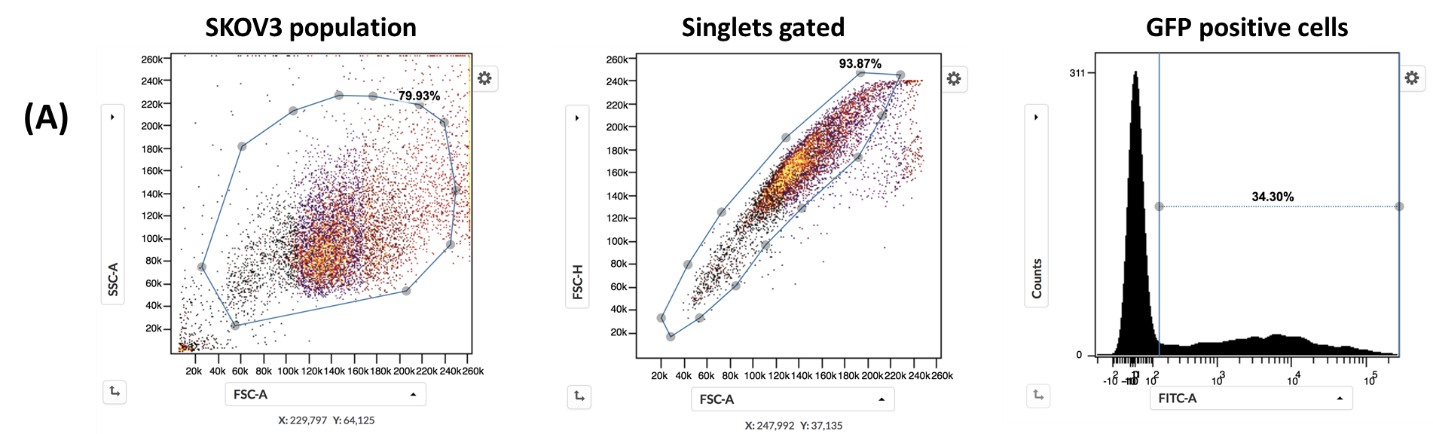


**Supplementary Figure S2**. The transfection efficiency of a GFP gene-containing plasmid into SKOV3 cells using different ratios of DNA to Lipofectamine or PEI transfection reagents. **(A)** The Cytobank workflow for GFP-positive cells determination. **(B)** The levels of GFP protein assessed on SKOV3 cells after transfection with different ratios of DNA: Lipo and DNA: PEI, respectively. A first gate on FSC-A *vs*. SSC-A dot plot was applied to select the intact cells; a second gate on FSC-A *vs.* FSC-H was applied to select only the singlets and a third marker gate was applied to separate between GFP fluorescence emission and autofluorescence.

**
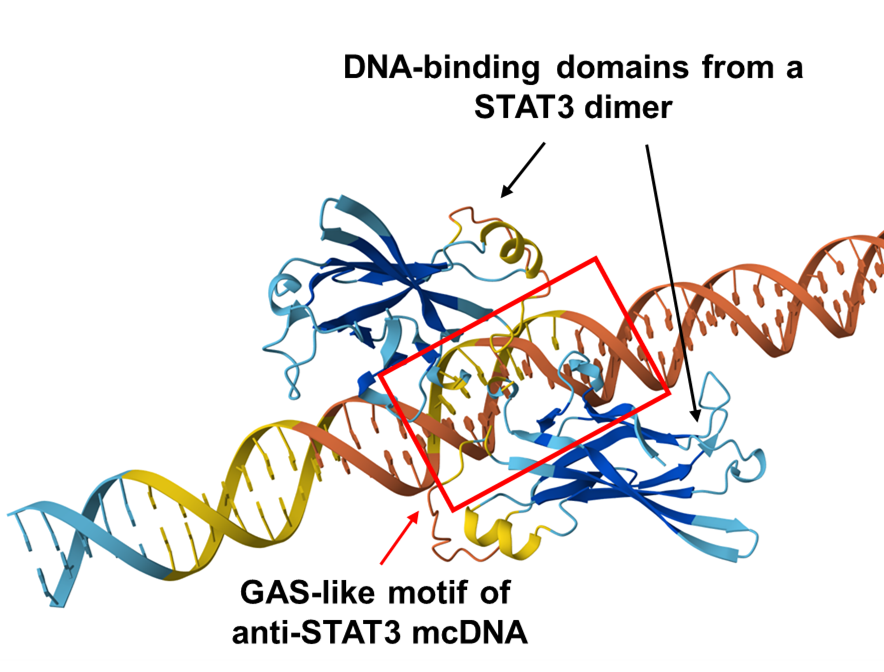
**

**Supplementary Figure S3**. 3D complex obtained in AlphaFold 3 Server. The black arrows mark the DNA-binding domains from a STAT3 dimer and the red arrow marks a GAS-like motif within the sequence of anti-STAT3 mcDNA. The DBDs of STAT3 proteins specifically recognize this consensus sequence and bind to it.

## Supplementary Tables

**Supplementary Table S1**. The specific primer sequences used for the amplification of *MCL1*, *PIM1* and *GAPDH* genes, in RT-qPCR.

| **Gene** | **Primer Sequences** |
| --- | --- |
| *MCL1* | For: 5’ ACCAAGAAAGCTGCATCGAACC 3’  Rev: 5’ CAGCACATTCCTGATGCCACC 3’ |
| *PIM1* | For: 5’ GCTCGGTCTACTCAGGCATCCG 3’  Rev: 5’ CCGAGCTCACCTTCTTCAGCAG 3’ |
| *GAPDH* | For: 5’ GTCTCCTCTGACTTCAACAGCG 3’  Rev: 5’ ACCACCCTGTTGCTGTAGCC 3’ |
